# Antibiotic use in pig farms at different levels of intensification—Farmers’ practices in northeastern Thailand

**DOI:** 10.1371/journal.pone.0243099

**Published:** 2020-12-11

**Authors:** Gunilla Ström Hallenberg, Jatesada Jiwakanon, Sunpetch Angkititrakul, Seri Kang-air, Kristina Osbjer, Kamonwan Lunha, Marianne Sunde, Josef D. Järhult, Thomas P. Van Boeckel, Karl M. Rich, Ulf Magnusson

**Affiliations:** 1 Department of Clinical Sciences, Swedish University of Agricultural Sciences, Uppsala, Sweden; 2 Research Group for Animal Health Technology, Khon Kaen University, Khon Kaen, Thailand; 3 Faculty of Veterinary Medicine, Khon Khon University, Khon Kaen, Thailand; 4 Section for Food Safety and AMR, Norwegian Veterinary Institute, Oslo, Norway; 5 Zoonosis Science Center, Department of Medical Sciences, Uppsala University, Uppsala, Sweden; 6 Institute for Environmental Decisions,–ETH Zürich, Zürich, Switzerland; 7 Center for Diseases Dynamics Economics and Policy, Washington, DC, United States of America; 8 Policies, Institutions, and Livelihoods Program, International Livestock Research Institute, West Africa Regional Office, Dakar, Senegal; Nitte University, INDIA

## Abstract

Understanding the patterns and drivers of antibiotic use in livestock is crucial for tailoring efficient incentives for responsible use of antibiotics. Here we compared routines for antibiotic use between pig farms of two different levels of intensification in Khon Kaen province in Thailand. Among the 113 family-owned small-scale farms (up to 50 sows) interviewed did 76% get advice from the pharmacy about how to use the antibiotics and 84% used it primarily for treating disease. Among the 51 medium-scale-farms (100–500 sows) belonging to two companies did 100% get advice about antibiotic use from the company’s veterinarian (P<0.0001) and 94% used antibiotics mostly as disease preventive measure (P<0.0001). In 2 small scale farms 3^rd^ generation cephalosporins, tylosin or colistin were used; antibiotics belonging to the group of highest priority critically important antimicrobials for human medicine. Enrofloxacin, belonging to the same group of antimicrobials, was used in 33% of the small-scale and 41% of the medium-scale farms. In the latter farms, the companies supplied 3–4 antibiotics belonging to different classes and those were the only antibiotics used in the farms. The median and mean estimated expenditure on antibiotics per sow was 4.8 USD (IQR = 5.8) for small-scale farms and 7 USD and 3.4 USD for the medium-scale farms belonging to the two respective companies. Our observations suggest to target the following areas when pig farming transitions from small-scale to medium-scale: (i) strengthening access to professional animal health services for all farmers, (ii) review of the competence and role of veterinary pharmacies in selling antibiotics and (iii) adjustment of farming company animal health protocols towards more medically rational use of antibiotics.

## Introduction

Antibiotic resistance (ABR) is foreseen to become a major health crisis of our time, threatening to leave us with restricted options to treat severe bacterial infections in the future. A decreased ability to treat these infections may result in serious consequences for human health and the health and productivity of livestock [1]. The emergence of ABR has partly been attributed to the irrational and inappropriate use of antibiotics in food-producing animals [2, 3]. This irrational use is often a consequence of lack of knowledge among farmers or inadequate practices among veterinarians [4] or of harmful economic incentives, as antibiotics may be used as a disease preventive measure or as growth promotors or for masking poor animal husbandry and biosecurity [5].

Current estimates based on sales volumes obtained from public records suggest that the total antibiotic use in food-producing animals exceeds that in humans on a global level [6]. Antimicrobial use in the livestock sector is expected to increase in the coming decades, predominately occurring in the in low- and middle-income countries and within the pig and poultry sectors [6]. This is a consequence of the growing demand for more varied diets including animal-source foods in these countries and that these two species are well-suited for intensification. As a response to this demand, farming systems are shifting towards more intensified large-scale systems, where animals are generally raised in larger groups and at higher densities [7–9]. These high densities demand good animal management, high biosecurity and adequate vaccination programs to prevent infections [10]. If these measures are not in place in intensive farming systems, antibiotics are prone to be more routinely used [11].

In Thailand, the standing population of pigs is estimated to be around 10 million heads [12]. Although the majority of farming households (>90%) are still smallholders with less than 50 pigs, the pig sector is intensifying with increasing number of pigs per farm [12, 13], trailing the intensification of chicken in the country [14]. Along with this intensification, farmers tend to prefer more commercial, exotic pig breeds, including Large White, Landrace, Duroc and crosses of these. Exotic breeds generally show higher growth rates than native breeds but are less adapted to the hot and humid climate in Southeast Asia and show lower resistance to endemic diseases, such as intestinal parasites and foot and mouth disease [15, 16]. Consequently, if the biosecurity is poor, higher animal densities with increased contact rates between genetically homogenous animals may create favorable conditions for disease transmission. In that respect, medium-sized pig farms with limited biosecurity may be hotspots for animal diseases and potentially also for extensive antibiotic use [17].

Past surveys have identified differences in the way antibiotics are used when comparing pig production at different levels of intensification. A positive association between a large farm size and antibiotic use has been reported from various regions in the world, including Thailand [18], China [19], Nigeria [20] and the Netherlands [21]. A high use of antibiotics is often reflected in high bacterial resistance levels on farm as well as on country level [22–24]. Thus, reducing the use and misuse of antibiotics is a crucial step towards limiting further emergence of ABR.

The objective of this study was to compare routines for antibiotic use between pig farms of two different levels of intensification in Khon Kaen province in Thailand. Understanding the patterns and drivers of antibiotic use in livestock in countries with emerging economies is crucial for tailoring efficient incentives for responsible use of antibiotics.

## Materials and methods

### Study setting

**Fig 1:**
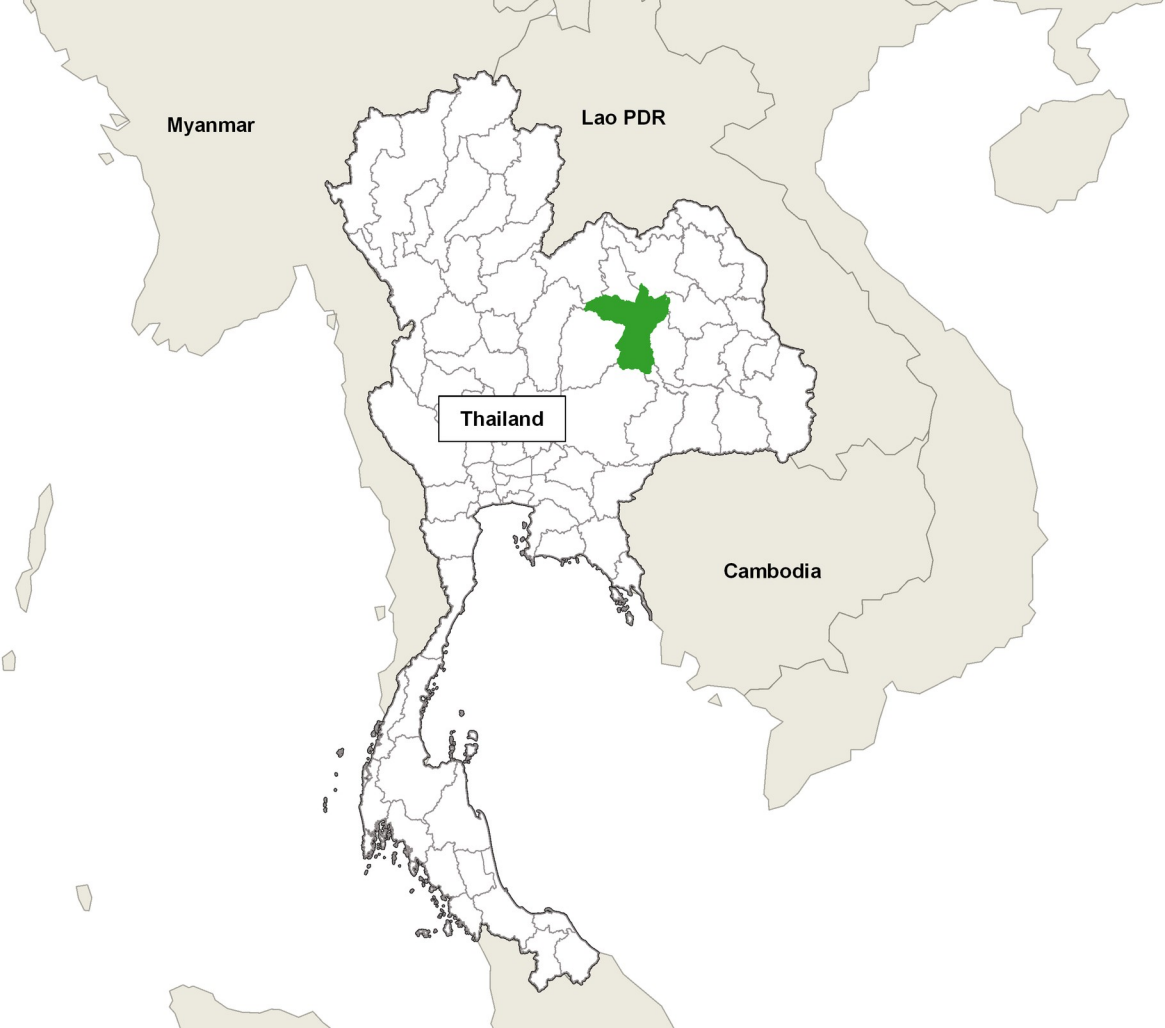
Study area. The pig farms in this study were all located in the Khon Kaen province in northeastern Thailand.

This cross-sectional study was conducted in the Khon Kaen province in northeastern Thailand from September to December, 2018 (Fig 1). The pig production in the province is dominated by small-scale farms that are owned and operated by families. Commercial farms in the province are mostly contract farms, i.e. farms that are owned by companies but operated by contract farmers. Regardless farm type, sows of Landrace x Large white was the most common breed.

Dissimilarities between pig farms of different levels of intensification with regards to farm and farmers characteristics, animal health and disease prevention, veterinary drug access, knowledge and use were studied. For this purpose, two categories of farms, based on the number of sows as this number fluctuates less over the year than the total number of pigs, were selected. Medium-scale farms were defined as farms keeping between 100 to 500 sows, while small-scale farms were defined as farms keeping less than 50 sows. These definitions were based on what has previously been used in other studies on pig farming in Thailand [13, 18]. All the medium-scale farms were contract breeding farms, belonging to either one of two large contracting companies. Fifty one medium-scale farms and 113 small-scale farms adding up to 164 farms in total were included in the study. All medium-scale farms in two districts (n = 51) were selected. The same number of small-scale farms were included from the same two districts, randomly selected from a list provided by the province veterinary officer. The additional 62 small-scale farms were selected in the same way from six surrounding districts without medium scale farms.

### Study procedure

A structured questionnaire, including questions on farm characteristics, disease history and routines for use of veterinary drugs based on the AMUSE-tool [25] was used and targeted at the person taking decisions about pig rearing at the farm (S1 Questionnaire). The questionnaire was developed in English and was translated into Thai. Interviews were carried out in Thai by two of the authors (JJ and SA), using a dialogue-based format. The answers were then translated back into English and recorded using Open Data Kit [26] an open-source smartphone platform that can be used to create electronic questionnaire forms for real-time data entry and management. In the ODK each farm was assigned a unique number.

### Expenses for antibiotics

To get an estimate of the yearly expenditure on antibiotics for sows, different approaches were followed for the small- and medium-scale farms. For small-scale farms, each farmer was asked to give an estimate of how much they spent on antibiotics (excluding use as feed additive) for sows in the last year. For medium-scale farms, a model (S1 File) was created for each of the two contracting companies to estimate the expenditure for all the farms belonging to the same company, as the farms within each company all shared similar routines with regard to antibiotic use, farm and animal management. The models were based on information provided by the veterinarian from each company regarding the costs for the routine administration of antibiotics as well as the estimated costs for antibiotics used for treatment of diseases. The model for each company was constructed as cost per sow and was then multiplied with the number of sows kept by each farm and applied for all farms within that company.

### Statistical analysis

Statistical analyses were conducted in SAS software 9.4 (SAS Institute Inc., Cary, NC). Descriptive statistics were calculated to define farm characteristics, disease history and to describe farmers’ practices and behaviors related to the use of veterinary drugs. Distributions for continuous variables (such as number of sows and age of the respondent) were tested for normality using the Shapiro-Wilks test. One-way analysis of variance (ANOVA) was used to test the difference in age of the respondent between the farm sizes. For the non-normally distributed variable ‘number of sows’, Kruskal Wallis test was used. Univariable logistic regression and Chi-square tests were used to examine possible associations between farm size, education levels, and management factors, such as routines related to veterinary drug use. Fisher’s exact test was used for frequencies of less than five. The statistical significance level was defined as a two-tailed P-value ≤ 0.05 for all models.

Since the expenditure on antibiotics for medium-scale farms was calculated based on a model, and farms within each company generally shared the same practices, associations and correlations between different factors were computed for small-scale farms only.

### Study approval and ethics

The study was conducted in accordance with the ethical standards of the institutional and/or national research committee and the Helsinki declaration. The protocol involving human participants and animals was approved by the Khon Kaen University Ethics Committee (Project ID: HE612268 and 0514.1.75/66 respectively). When being asked to participate in the study the farmers were informed about the purpose of the study and given guarantee that their identity should not be disclosed outside the research team. Verbal consent was then obtained and witnessed by at least two in the research team and at least one other person at the farm. Recording the interview served as documentation that the informed consent was obtained.

## Results

### Farm characteristics

The person interviewed was the owner of the pigs at 99% of the farms. There was a significant difference in age of the respondent between farms of different sizes, where farmers operating medium-scale farms were younger than farmers operating small-scale farms (P = 0.0002) (Table 1). There was a significant difference in level of education attained by the respondents, where medium-scale farmers had attained higher education compared with small-scale farmers. When included in the same model, after excluding interactions between the variables, both age (P = 0.012) and education level (P = 0.047) were significantly associated with farm size, indicating that medium-scale farms were operated by younger and more educated farmers.

**Table 1:** Farm characteristics of the small- (n = 113) and medium-scale farms (n = 51) included in the study (Khon Kaen, 2018).

	Category	Small-scale	Medium-scale	P value
		% (n)	% (n)	
Sex of respondent	Male	50.4 (57)	60.8 (31)	ns
	Female	49.6 (56)	39.2 (20)	
Age respondent	Mean	54.1	48.2	0.0002
	SD	9.7	7.9	
Education level respondent	Primary education (P1-P7)	66.4 (75)	39.2 (20)	0.0011
	Secondary school (S1-S6)	26.6 (30)	37.3 (19)	ns
	University degree	7.1 (8)	23.5 (12)	0.0029
Main income source	Crop farming	37.2 (42)	0 (0)	<0.0001
	Pig farming	44.3 (50)	100 (51)	<0.0001
	Salaried employment	2.7 (3)	0 (0)	ns
	Self-employed off farm	10.6 (12)	0 (0)	0.0188
	Casual laboring	2.6 (3)	0 (0)	ns
	Other	2.6 (3)	0 (0)	ns
Hired workers on the farm	Yes	0.9 (1)	29.4 (15)	<0.0001
	No	99.1 (112)	70.6 (36)	
Number of sows	Mean	4	202	<0.0001
	SD	6.0	52.3	
Main responsibility for the pigs	Male household head	49.6 (56)	60.8 (31)	ns
	Female household head	48.7 (55)	35.3 (18)	ns
	Son	0.9 (1)	0 (0)	ns
	Employee	0.9 (1)	3.9 (2)	ns

All medium-scale farmers listed pig farming as their main income source, while the small-scale farmers had more diverse income sources besides pig farming, including crop farming and employments outside the farm (Table 1). Furthermore, all farmers operating medium-scale farms stated that the income from pig farming constituted half or more of their household’s income, whereas this was stated by 63% the small-scale farmers (P<0.0001).

The mean number of sows kept by small-scale farms at the time of visit was 4 (SD = 6), while corresponding figure was 202 sows (SD = 52) for medium-scale farms (Table 1). All farms kept their pigs in enclosures.

### Animal health and disease prevention

All medium-scale farmers reported to have experienced diseases in their pigs in the past 12 months, whereas this was reported by 74% of the small-scale farmers (Table 2). The disease most commonly reported by both small- and medium-scale farms were diseases related to the digestive tract (e.g. diarrhea). Almost 18% of the small-scale farmers reported other diseases than the ones pre-listed in the questionnaire, most commonly fever and lameness. Farmers were also asked whether they had experienced diseases in their pigs within the past two weeks, which was the case for the majority of the medium-scale farmers (94%) but only for a quarter of the small-scale farmers (23%) (P<0.0001). All medium-scale farmers reported that this disease was diagnosed by company staff instead of themselves. The majority of the small-scale farmers (85%) had not asked any advice for diagnosis.

**Table 2:** Farmers’ responses on animal health and disease prevention (Khon Kaen, 2018).

	Category	Small-scale	Medium-scale	P value
		% (n)	% (n)	
What was the main disease problem during the last 12 months?	Respiratory	2.7 (3)	0 (0)	ns
	Digestive tract	45.1 (51)	88.2 (45)	<0.0001
	Reproductive	5.3 (6)	0 (0)	ns
	Skin disease/wounds	0.9 (1)	0 (0)	ns
	Neurological signs	2.6 (3)	0 (0)	ns
	No disease	25.7 (29)	0 (0)	<0.0001
	Other	17.7 (20)	11.8 (6)	ns
Have you had any diseases among the pigs in the past 2 weeks?	Yes	23.0 (26)	94.1 (48)	<0.0001
	No	77.0 (87)	5.9 (3)	
What disease?	Respiratory	2.7 (3)	2.0 (1)	ns
	Digestive tract	15.9 (18)	94.1 (48)	<0.0001
	Fever	2.7 (3)	27.5 (14)	<0.0001
Was the disease diagnosed by others than yourself?	Yes	15.4 (4)	100 (48)	<0.0001
	No	84.6 (22)	0 (0)	
If yes, who diagnosed the disease?	Company staff	25 (1)	100 (48)	0.0002
	Animal health worker	25 (1)	0 (0)	ns
	Government veterinarian	50 (2)	0 (0)	0.005
Do you do anything in order to protect the pigs against disease?	Yes	84.1 (95)	100 (51)	0.0009
	No	15.9 (18)	0 (0)	
What kind of protective measure?	Fences around farm*	14.2 (16)	100 (51)	<0.0001
	Medicated feed	0.9 (1)	47.1 (24)	<0.0001
	Vaccination	75.2 (85)	100 (51)	<0.0001
Are you involved in any animal health program (e.g. vaccination program)?	Yes	77.9 (88)	100 (51)	<0.0001
	No	22.1 (25)	0 (0)	
What do you do in response to disease problems?	Use traditional medicine	6.2 (7)	0 (0)	ns
	Use medicine from veterinary drug store (self-bought)	72.6 (82)	0 (0)	<0.0001
	Consult traditional healer	1.8 (2)	0 (0)	ns
	Consult animal health worker	15.9 (18)	0 (0)	0.0009
	Consult private veterinarian	4.4 (5)	100 (51)	<0.0001
	Consult government veterinarian	5.3 (6)	0 (0)	ns
	I do nothing	6.2 (7)	0 (0)	ns

*For small-scale farms, ‘fences’ generally referred to metal wire or similar, while at medium-scale farms it generally referred to solid walls.

All medium-scale farms were contract farms, they shared similar routines with regard to farm and animal management through support from one of the two contractors (Table 2). All medium-scale farmers reported to consult a veterinarian if the pigs showed symptoms of disease, whereas consultation with a veterinarian was less frequent among small-scale farmers: 73% reported self-administration of medicines bought at the veterinary drug store.

### Use of veterinary drugs

All medium-scale farmers reported to have easy access to veterinary drugs, compared with 78% of the small-scale farmers (Table 3). All medium-scale farmers reported to receive the drugs and ask advice from either the veterinarian or the staff from their respective contracting company, while 76% of the small-scale farmers reported to ask advice from the staff in the pharmacy or veterinary drug store where drugs were bought. Of the medium-scale farms, all farms belonging to one of the companies reported to use feed that contained antibiotics, whereas this type of feed was used by only 13% of the farms belonging to the other company. Only one of the small-scale farms reported usage of a feed that contained antibiotics.

**Table 3:** Reported access to veterinary drugs and advice related to veterinary drug use as reported by the farmers (Khon Kaen, 2018).

	Category	Small-scale	Medium-scale	P value
		% (n)	% (n)	
Easy access to veterinary drugs	Yes	77.9 (88)	100 (51)	<0.0001
	No	22.1 (25)	0 (0)	
Do you ask advice on how to use veterinary drugs?	Yes	88.6 (78)	100 (51)	0.0136
	No	11.4 (10)	0 (0)	
If yes, via which channel do you get the advice?	Veterinarian	4.6 (4)	92.2 (47)	<0.0001
	Animal health workers	8.0 (7)	0 (0)	0.0472
	Pharmacy/drug store	76.1 (67)	0 (0)	<0.0001
	Other farmers	5.7 (5)	0 (0)	Ns
	Drug packages	1.1 (1)	0 (0)	Ns
	Company staff	0 (0)	100 (51)	<0.0001

All medium-scale farmers reported to have used veterinary drugs within the past month, while 70% of the small-scale farmers reported this practice (Table 4). The mean number of different antibiotics used was 1.1 (SD = 0.99) by small-scale farms and 3.4 (SD = 0.49) by medium-scale farms. All medium-scale farms reported to have used between three and four drugs recently, following the company guidelines, whereas nine (8%) of the small-scale farms reported using three drugs or more. The most common drugs used within the past month were enrofloxacin, penicillin/streptomycin combination and amoxicillin for the small-scale farms, and gentamicin, penicillin/streptomycin combination and amoxicillin for the medium-scale farms (Table 5).

**Table 4:** Reported practices related to the administration of the most commonly used drug in the past month (Khon Kaen, 2018).

	Category	Small-scale	Medium-scale	P value
		% (n)	% (n)	
Have you used veterinary drugs in the past month?	Yes	69.9 (79)	100 (51)	<0.0001
	No	30.1 (34)	0 (0)	
If yes, how did you access the drugs?	Veterinary drug store	97.5 (77)	0 (0)	<0.0001
	Company	0 (0)	100 (51)	<0.0001
	Animal health workers	5.1 (5)	0 (0)	Ns
	Private veterinarians	3.8 (3)	0 (0)	Ns
	Feed providers	3.8 (3)	0 (0)	Ns
Reason for drug use	Treatment of diseases	83.5 (66)	47.1 (24)	<0.0001
	Prevention of diseases	27.9 (22)	94.1 (48)	<0.0001

**Table 5:** Antibiotics used within the past month as reported by the small-scale (n = 113) and medium-scale (n = 51) farmers (Khon Kaen, 2018).

Drug class	Antibiotic	Small-scale	Medium-scale^1^
		% (n)	% (n)
Aminoglycocides	Gentamicin	7.1 (8)	100 (51)
	Kanamycin	5.3 (6)	0 (0)
β-lactams	Amoxicillin	10.6 (12)	58.8 (30)
	Penicillin	1.8 (2)	0 (0)
3^rd^ gen cephalosporins	Ceftriaxone^2^	0.9 (1)	0 (0)
3^rd^ gen cephalosporins	Ceftiofur^2^	1.8 (2)	0 (0)
1^st^ gen cephalosporins	Cephalexin	0 (0)	41.2 (21)
Fluoroquinolones	Enrofloxacin^2^	32.7 (37)	41.2 (21)
Lincosamides	Lincomycin	0.9 (1)	0 (0)
Macrolides	Tylosin^2^	0.9 (1)	0 (0)
Tetracyclines	Chlortetracycline	0.9 (1)	0 (0)
	Oxytetracycline	0.9 (1)	0 (0)
Pleuromutilins	Tiamulin	2.7 (3)	0 (0)
Polymyxines	Colistin^2^	1.8 (2)	0 (0)
β-lactams/Aminoglycosides	Penicillin-streptomycin	17.7 (20)	100 (51)
Lincosamides/Aminoglycosides	Lincomycin-streptomycin	0.9 (1)	0 (0)
Tetracyclines/Aminoglycosides	Oxytetracycline-streptomycin	0.9 (1)	0 (0)

^1^In medium-scale farms, the companies supplied 3–4 different antibiotics and those were the only antibiotics used in the farms.

^2^ Antibiotics that are on the list of “Highest Priority Critically Important Antimicrobials”for human medicine [27].

There was a significant difference in the reason why drugs had been used within the past month, where the majority of the small-scale farmers reported the use to be related to treatment of diseases (84%) (Table 4), using the drugs until the animal was cured. Medium-scale farmers, on the other hand, had most commonly used the drugs as a preventive measure (94%) (P<0.0001), most often injecting the drugs to individual animals at a single occasion The practice to use antibiotics only once in the medium-scale farms was majorly referring to injections of healthy sows after farrowing, which was also included in the company treatment guidelines.

Farmers were asked whether they had experienced any situation where the drugs for the intended treatment had not worked (i.e. the animal had not recovered). Twenty-one percent of the small-scale farmers and 65% of the medium-scale farmers reported that treatment failure sometimes occurred. However, the majority of the small-scale farmers (77%) and 35% of the medium-scale farmers reported to never have experienced treatment failure.

Regarding the handing of expired drugs, all medium-scale farmers reported to return the drugs to their company, while small-scale farmers most commonly (94%) stated that they ‘disposed of’ these drugs.

All respondents, regardless of farm size, were aware of that vaccines were used for prevention of diseases and that antibiotics could be used for treatment of diseases. However, only 18% of the small-scale farmers responded that antibiotics could be used also as a preventive measure, while this was stated by 94% of the medium-scale farmers (P<0.0001; OR = 74; CI = 21–263).

### Expenses for antibiotics

The median estimated expenditure on antibiotics per sow was 4.8 USD (IQR = 5.8) for small-scale farms. For medium-scale farms, the calculated mean expenditures were 7.0 and 3.4 USD for the two respective companies.

For the small-scale farms, associations between estimated antibiotic expenditure and farmers’ characteristics and practices, respectively, were investigated. There were no significant associations between the annual cost for antibiotics and the sex or education level of the farmer. However, farmers that reported to have experienced diseases in the past 12 months and within the past 2 weeks had significantly higher estimated costs for antibiotics compared with farmers that had not experienced diseases (P<0.0001 and P = 0.005, respectively). Farmers that reported to have used veterinary drugs in the past month had higher estimated annual costs for antibiotics (P = 0.02) and the number of drugs used in the past month was positively correlated with higher costs (P = 0.0003). Farmers that reported never to have experienced a situation where an antibiotic did not work had significantly lower estimated annual costs for antibiotics (P = 0.0009). There were no significant associations between estimated antibiotic expenditure and having access to animal health services or among farmers reporting to seek advice on how to use veterinary drugs.

Among the farmers that reported to have used drugs within the past month, there were significantly lower estimated annual costs by farmers that reported to have used the most commonly used drug, which varied between farms, as a preventive measure (P = 0.0255). In contrast, farmers that reported to have used this drug for treatment had higher estimated yearly costs for antibiotics (P = 0.0015). Farmers that reported to administer the drug until the animal was cured reported higher costs for antibiotics, compared with farmers that reported to administer the antibiotics as a one-time event (P = 0.0003).

## Discussion

The antibiotic use in the livestock sector is expected to increase in the coming decades, partly as a consequence of livestock systems shifting from small-scale extensive to more intensive large-scale farming in low- and middle-income countries [6, 7]. This increase could exacerbate the rise of ABR in animals raised for food animals in LMICs [28]. Therefore, it is important to understand how pig farmers’ practices related to antibiotic use may change following the shift from extensive to intensive farming. This will give the option to promote effective means to mitigate the ABR emergence originating from the livestock sector. In this cross-sectional study we investigated different management routines related to antibiotic use at pig farms in small and medium size farms in northeastern Thailand, and compared estimated costs for antibiotics on these farms.

First, there was a fundamental difference in the operation of small-scale and medium-scale farms. The medium-scale farms were all contract farms belonging to two large companies whereas the small-scale farms were privately owned. Consequently, the differences reported in this study may not exclusively be related to farm size, but also to ownership of the farm. The contract farms were obliged to adhere to the guidelines of their respective company and received all input (e.g. feed and medicines) from their company. Through the company, farmers had access to various support, including veterinary services and vaccination programs. In contrast, only 10% of all small-scale farmers reported to consult a veterinarian if an animal became sick. These farms were generally operated by older and less educated farmers and equally by women and men, who were often involved in a variety of income-generating activities, besides pig raising. The majority of the small-scale farmers reported to purchase antibiotics directly from their local veterinary drug store when the pigs were sick and to ask advice from the staff working in the store. In Thailand, the Code of Practice for Control of the Use of Veterinary Drugs states that a qualified veterinarian is required to be responsible for veterinary drug stores and pharmacies and is mandated to supervise the staff, meaning that the daily operation is not necessarily run by a trained veterinarian [29]. It is likely that small-scale farmers in similar settings around the world lack easy access to the veterinary services necessary for deciding on an appropriate antibiotic treatment and hence solely rely on their own judgement or on advice from vendors, who may not necessarily have received sufficient training or who may have vested interest. For successful future interventions in improving the antibiotic use in small-scale farming, not only farmers should be targeted but also the veterinary drug stores. This approach has previously been successfully used to reduce the misuse of human medicines available over the counter [30].

Previous studies have shown a positive association between farm size and the use of antibiotics [20, 21], potentially due to a greater disease pressure on farms with a high density of animals. However, it has been reported that this positive relationship might only apply when considering farms keeping up to around 500 pigs [19]. It is suggested that it is these medium-sized farms that are the heaviest users of antibiotics since larger pig farms use more vaccinations and biosecurity measures, such as segregation of animals and frequent cleaning of the premises. These differences were proposed to potentially result from larger farms having more capital to invest in biosecurity infrastructure and veterinary services compared with smaller farms. In the current study we found that the medium-scale farmers did have better access to veterinary services and infrastructure compared with the small-scale farmers. One may also speculate that the cost for pharmaceuticals, including antibiotics, were lower for the medium- scale contract farms and thus influence the use of antibiotics. However, as the data presented refer to the estimated costs and not the recorded amount of antibiotics used, we cannot make solid comparisons between the small- and medium-scale farms.

We found that medium-scale farms used antibiotics mainly for disease prevention whereas small-scale farms used them for treatment. Other studies have reported similar findings, where antibiotic use within small-scale extensive livestock production is mostly for therapeutic purposes rather than for disease prevention or growth promotion [31, 32]. In the present study, only one-fifth of the small-scale farmers reported to be aware of the disease-preventive effect of antibiotics, compared with almost all medium-scale farmers, which might partly explain the usage differences. This is also possibly influenced by the difference in education level and access to veterinary service between small- and medium-scale farmers. Lekagul and co-workers [31] found that the practice of using antibiotics as a disease preventive measure in pig production was associated with more experienced farmers. Thus, simply increasing farmer knowledge on antibiotics might not be sufficient to improve usage routines on the farms but should be coupled with education on good animal husbandry and farm management, including housing, feed and water quality, vaccination and biosecurity. Notably, the finding in the current study do also identify veterinary drugstore retailers and company veterinarians as target groups besides the farmers for increasing awareness and knowledge. The funding for, and thus the access to, such education activities or extension service has been a challenge around the world for years [33].

The majority of the medium-scale farms reported to use medicated feed for the pigs, while this was reported by only one of the small-scale farms. The latter likely because of the extra cost for prefabricate feed with medication. The contract (medium-scale) farms had to adhere to their respective company guidelines regarding feed and other farm input, but it seems that some (13%) of the contract farmers were unaware of that the feed contained antibiotics as it was not written on the feed package.

The annual cost of antibiotics used for sows was estimated for all the participating farms. According to these estimates there were large individual variations among small-scale farms (median: 4.8 USD; IQR: 5.8 USD) but also between the two companies (mean: 7 and 3.4 USD). The latter could partly be explained by that one of the companies used two types of antibiotics for routine disease prevention while the other company used three antibiotics. However, it is remarkable that the mean estimated cost for the small-scale farms was about the same as for the medium-scale farms, despite the fact that medium-scale farms performed extensive routine herd treatment. These estimates should be interpreted with caution since they are based on self-reported estimates and generally just few small-scale farmers keep records of the types and the amount of antibiotics used, despite that it is recommended by the governmental agricultural standards [29]. Even so, possibly the price for each dose of antibiotics were cheaper for the medium-scale farmers as they belonged to companies that benefitted from buying large quantities of medicines without middle-men such as veterinary drug shops.

The antibiotics used, regardless farm-type, are acting on a broad spectrum of bacteria and some are on the on the list of “Highest Priority Critically Important Antimicrobials”for human medicine [27]. One such antibiotic, enrofloxacin, was commonly used in small scale-farms and part of the antibiotic scheme in one of the companies owing the medium-scale farms. Colistin, 3^rd^ generation cephalosporins or tylosin, also on that list, were just used in two of the small-scale farms, but not on the medium-scale farms. At large, the selection of the kinds of antibiotic used reflects limited concerns about public health risks.

Small-scale farmers that reported to have used veterinary drugs in the past month had significantly higher estimated annual costs for antibiotics and the number of drugs used in the past month was correlated with higher costs. This finding could suggest that these farms use in general more antibiotics, although the responses could be influenced by recall bias. Interestingly, small-scale farmers that reported never to have experienced treatment failure had significantly lower estimated costs for antibiotics. It is possible that these farmers, as a result of their potential lower drug use, did not encounter any problem with resistance on their farms, though this interpretation is based on rather uncertain self-reported data. Conversely, it is likely farmers with emerging ABR on their farms that are experiencing increasing costs for antibiotics due to reduced efficacy of the antibiotics used. Moreover, we found that small-scale farmers that reported to have used the most common drugs for prevention had lower estimated costs for antibiotics while the small-scale farmers that had used the drug for treatment had higher costs. It is possible that farmers that rely on antibiotics for disease prevention, instead of investing in better farm management and biosecurity, are experiencing more immediate savings since antibiotics are comparatively cheap [4]. Since many small-scale farmers have various income sources and might only pursue pig raising in times of profit, they are likely to prioritize short term cuts in costs and might be reluctant to make more comprehensive investments. These findings highlight the difficulties in changing current practices related to antibiotic use within small-scale farming.

## Conclusion

We found that medium-scale, more intensive, farming systems had access to professional veterinary services that handled the antibiotic use through their contracting company, whereas the small-scale farms had limited access to veterinary advices and had to rely on veterinary pharmacies regarding the use of antibiotics. A major reason for this was likely the difference in ownership of the farms, companies and family, respectively, disclosing the need for publicly accessible veterinary services. It should be noted that the small-scale farms rarely used antibiotics for disease prevention, whereas this was a common practice in the medium-scale farms. Thus, in order to refine the use of antibiotics in pig farming in emerging economies, the observations from this study suggest to target the following areas when pig farming transitions from small-scale to medium-scale: (i) strengthening access to professional animal health services for all farmers, (ii) review of the competence and role of veterinary pharmacies in selling antibiotics and (iii) adjustment of farming company animal health protocols towards more medically rational use of antibiotics.

## Supporting information

S1 Questionnaire (DOCX)

S1 File (TXT)

S1 Dataset (TXT)
