# Supplementary material for: Antibiotic use in pig farms at different levels of intensification—Farmers’ practices in northeastern Thailand
Source: PLoS One. 2020 Dec 11;15(12):e0243099. doi: 10.1371/journal.pone.0243099 (PMC7732346; doi:10.1371/journal.pone.0243099)
Supplement: S1 Questionnaire — (DOCX) [file pone.0243099.s001.docx]

| **Criteria for selecting respondent: person who plays a major role in the management of livestock information ON ENUMERATION** | | | |
| --- | --- | --- | --- |
| 1. Questionnaire ID | |  | |
| 1. Date of Survey (DD/MM/YYYY) | |  | |
| 1. Enumerator’s name (First Name and Last Name) | | List of names of enumerators | |
| 1. Interview done via interpreter | | 🞏1=yes  🞏2=now | |
| 1. Enumerator’s sex | | 🞏1=Male, (If list available then this should be automatically filed)  🞏2=Female | |
| 1. Time interview started (HH:MM) | | Will be automatically generated by the tablets | |
| 1. Time interview ended (HH:MM) | | Will be automatically generated by the tablets | |
| 1. Consent received (signature on form if literate) | | 🞏1=yes  🞏2=no | |
| **FARM BASICS AND LOCATION** | | | |
| 1. District | | List of all districts pre-coded | |
| 1. Sub-county | | List of all sub counties pre-coded | |
| 1. Parish | | List of all parishes pre-coded | |
| 1. Village | | List of all village pre-coded | |
| 1. GPS Coordinates | | Will be automatically generated by the tablets | |
| **HOUSEHOLD DEMOGRAPHICS** | | | |
| 1. Sex of the Household head | | 🞏1=Male  🞏2=Female | |
| 1. Sex of the respondent (if other than household head) | | 🞏1=Male  🞏2=Female | |
| 1. Age of respondent (years) | | …………………. | |
| 1. Role of the respondent in relation to livestock (multiple answers possible) | | 🞏1 Management  🞏2 Marketing  🞏3 Owner  🞏4 None  🞏 5 other | |
| 1. What is the main source of income for the household? (Mark one) | | 🞏 1 crop farming  🞏 2 cattle keeping  🞏 3 pig keeping (inc. sales)  🞏 4 small ruminant keeping  🞏 5 poultry keeping  🞏 6 salaried employment  🞏 7 self-employed-off farm  🞏 8 casual laboring  🞏 9 Boda-boda  🞏 10 other (specify)…………………………………………….. | |
| 1. Livestock contributes to | | 🞏 1 To half or more of the household’s income  🞏 2 To less than half of the household’s income  🞏 3 Does not contribute to the household income | |
| 1. What is the education level of the **respondent**? | | 🞏 1 Never went to school  🞏 2 Non-formal education (years)………………………  🞏 3 Primary education (P1-P7)  🞏 4 Secondary school (S1-S6)  🞏 5 Vocational training (specify)………………………….  🞏 6 University degree (undergraduate)  🞏 7 Adult literacy | |
| 1. Do you have hired workers on the farm | 🞏 Yes 1  🞏 No, family members only 2 | | |
| **FARM CHARACTERISTICS** | | | |
| 1. What livestock do you have? (filter question) | | 🞏1Cattle (beef, dairy)  🞏2Small ruminants  🞏3Poultry  🞏4Pigs  🞏5Equine  🞏6Camel | |
| 1. Who has the **main** responsibilty (for each species) | | | |
| 🞏 1 Household head (man) | | | |
| 🞏 2 Household head (woman) | | | |
| 🞏 3 Joint responsibility (Couple | | | |
| 🞏 4 Daughter | | | |
| 🞏 5 Son | | | |
| 🞏 6 Employee | | | |
| 🞏 7 Other (specify) | | | |
| 1. Herd flock size (mumber of animals for each species) | | | |
| 1. Poultry | | Number………………………. | |
| Equines | | Number….. | |
| 1. Cattle | | Adult males (>2 years) |  |
|  |  | Adult females (>2 years) |  |
|  |  | Calves/heifers |  |
| 1. Pigs | | Sows |  |
|  |  | Boars |  |
|  |  | Growers/fatteners |  |
|  |  | Piglets (<3 moths) |  |
| 1. Small ruminants | | Males (>1 year) |  |
|  |  | Females (>1 year) |  |
|  |  | Young |  |
| 1. Camels | |  | |

| 1. Characteristics of livestock production systems (single choice/most common practice throughout the year) | | |
| --- | --- | --- |
| **1. PIGS** | | 🞏 1= free-range  🞏 2= tethered  🞏 3= housed |
| **2. POULTRY** | | 🞏 1= free-range  🞏 2= housed |
| **3. Cattle** | 🞏Beef | 🞏1 = Zero grazing  🞏2 = Fenced individual farm grazing  🞏3 = Communal grazing  🞏4 = Pastoral |
|  | 🞏 Dairy | 🞏1 = Zero grazing  🞏2 = Fenced individual farm grazing  🞏3 = Communal grazing  🞏4 = Pastoral |
| 4. Small ruminants | | 🞏1 = Zero grazing  🞏2 = Fenced individual farm grazing  🞏3 = Communal grazing  🞏4 = Pastoral |
| 5. Equines | | 🞏1 = Zero grazing  🞏2 = Fenced individual farm grazing  🞏3 = Communal grazing  🞏4 = Pastoral |
| 6. Camels | | 🞏1 = Zero grazing  🞏2 = Fenced individual farm grazing  🞏3 = Communal grazing  🞏4 = Pastoral |

| 1. Do you sell milk | 🞏 Yes 1  🞏 No 2 | | | | | | | | | | | |
| --- | --- | --- | --- | --- | --- | --- | --- | --- | --- | --- | --- | --- |
| 1. Which period of the year do you regularly sell milk? (multiple choices are allowed for the months) | Throughout the year 🞏 | | | | | | | | | | | |
|  | Seasonal (use calendar below) 🞏 | | | | | | | | | | | |
|  | J | F | M | A | M | J | J | A | S | O | N | D |
|  |  |  |  |  |  |  |  |  |  |  |  |  |
| 1. Do you sell eggs (multiple choices are allowed for the months) | 🞏 Yes 1  🞏 No 2 | | | | | | | | | | | |
| 1. Which period of the year do you regularly sell eggs? (multiple choices are allowed for the months)for the months | Throughout the year 🞏 | | | | | | | | | | | |
|  | Seasonal (use calendar below) 🞏 | | | | | | | | | | | |
|  | J | F | M | A | M | J | J | A | S | O | N | D |
|  |  |  |  |  |  |  |  |  |  |  |  |  |
| 1. Do you sell live animals ? | 🞏 Yes 1  🞏 No 2 | | | | | | | | | | | |

| 1. If yes, which species? Indicate during which months of the year you sell live animals (multiple choices are allowed for the months) | 🞏1 **Pigs**  Throughout the year 🞏  Seasonal (use calendar below) 🞏 | | | | | | | | | | | | | | | | | | | | | | |
| --- | --- | --- | --- | --- | --- | --- | --- | --- | --- | --- | --- | --- | --- | --- | --- | --- | --- | --- | --- | --- | --- | --- | --- |
|  | J | F | | M | | A | | M | | J | | J | | A | | S | | O | | N | | D | |
|  |  |  | |  | |  | |  | |  | |  | |  | |  | |  | |  | |  | |
|  | 🞏2 **Poultry**  Throughout the year 🞏  Seasonal (use calendar below) 🞏 | | | | | | | | | | | | | | | | | | | | | | |
|  | J | F | | M | | A | | M | | J | | J | | A | | S | | O | | N | | D | |
|  |  |  | |  | |  | |  | |  | |  | |  | |  | |  | |  | |  | |
|  | 🞏3 **Cattle**  Throughout the year 🞏  Seasonal (use calendar below) 🞏 | | | | | | | | | | | | | | | | | | | | | | |
|  | J | F | | M | | A | | M | | J | | J | | A | | S | | O | | N | | D | |
|  |  |  | |  | |  | |  | |  | |  | |  | |  | |  | |  | |  | |
|  | 🞏4 Small ruminants  Throughout the year 🞏  Seasonal (use calendar below) 🞏 | | | | | | | | | | | | | | | | | | | | | | |
|  | J | F | | M | | A | | M | | J | | J | | A | | S | | O | | N | | D | |
|  |  |  | |  | |  | |  | |  | |  | |  | |  | |  | |  | |  | |
|  | 🞏5 **Equines**  Throughout the year 🞏  Seasonal (use calendar below) 🞏 | | | | | | | | | | | | | | | | | | | | | | |
|  | J | | F | | M | | A | | M | | J | | J | | A | | S | | O | | N | | D |
|  |  | |  | |  | |  | |  | |  | |  | |  | |  | |  | |  | |  |
|  | 🞏6 **Camel**  Throughout the year 🞏  Seasonal (use calendar below) 🞏 | | | | | | | | | | | | | | | | | | | | | | |
|  | J | F | | M | | A | | M | | J | | J | | A | | S | | O | | N | | D | |
|  |  |  | |  | |  | |  | |  | |  | |  | |  | |  | |  | |  | |

| **MANAGEMENT OF MANURE, FEED AND WATER** | | | | | | | | | | | | |
| --- | --- | --- | --- | --- | --- | --- | --- | --- | --- | --- | --- | --- |
| 1. Manure management (by species), tick the **most common** option for each species (single choice). | | | | | | | | | | | | |
| Activity | 1.Cattle | | 2.Small ruminants | | 3. Equines | | 4. Poultry | | 5. Pigs | | 6. Camel | |
| 1. Leave on farm, do nothing |  | |  | |  | |  | |  | |  | |
| 1. Discard into environment |  | |  | |  | |  | |  | |  | |
| 1. Open air |  | |  | |  | |  | |  | |  | |
| 1. Used as fertilizer |  | |  | |  | |  | |  | |  | |
| 1. Use for fuel (incl. biogas) |  | |  | |  | |  | |  | |  | |
| 1. Sold for cash |  | |  | |  | |  | |  | |  | |
| 1. Taken by other farmers |  | |  | |  | |  | |  | |  | |
| 1. Other (specify) |  | |  | |  | |  | |  | |  | |
| 1. Feed products used per species (mulpile answers per species possible) , tick | | | | | | | | | | | | |
| Type of feed | | 1.Cattle | | 2.Small ruminants | | 3. Equines | | 4. Poultry | | 5. Pigs | | 6. Camel |
| Pasture/scavenging | |  | |  | |  | |  | |  | |  |
| Waste (household/restaurant, etc) | |  | |  | |  | |  | |  | |  |
| grains/crop residues | |  | |  | |  | |  | |  | |  |
| Feed mixed at farm | |  | |  | |  | |  | |  | |  |
| Commercial/pre-mix | |  | |  | |  | |  | |  | |  |
| Other | |  | |  | |  | |  | |  | |  |

| **ANIMAL HEALTH AND DISEASE PREVENTION** | | | | | | | | | | | | | | | | | | | | | | | | | | | | | |
| --- | --- | --- | --- | --- | --- | --- | --- | --- | --- | --- | --- | --- | --- | --- | --- | --- | --- | --- | --- | --- | --- | --- | --- | --- | --- | --- | --- | --- | --- |
|  | | | | | | | | | | | | | | | | | | | | | | | | | | | | | |
| 1. What was the **main animal disease problem** during the last 12 months (one disease per species)-if the farmers says FEVER, probe for more clinical signs because fever is common for most diseases | | | | | | | | | | | | | | | | | | | | | | | | | | | | | |
| Clinical signs | 1.Cattle | | 2.Goats | | | | | 3. Sheep | | | | | 4. Poultry | | | | | | 5. Pigs | | | | 6. Equines | | | | | 7. Camel | |
| a) Respiratory |  | |  | | | | |  | | | | |  | | | | | |  | | | |  | | | | |  | |
| b) Digestive tract/intestinal |  | |  | | | | |  | | | | |  | | | | | |  | | | |  | | | | |  | |
| c) Reproductive |  | |  | | | | |  | | | | |  | | | | | |  | | | |  | | | | |  | |
| d) Mastitis |  | |  | | | | |  | | | | |  | | | | | |  | | | |  | | | | |  | |
| e) Sudden death |  | |  | | | | |  | | | | |  | | | | | |  | | | |  | | | | |  | |
| f) Skin disease/wounds |  | |  | | | | |  | | | | |  | | | | | |  | | | |  | | | | |  | |
| g) External parasites |  | |  | | | | |  | | | | |  | | | | | |  | | | |  | | | | |  | |
| h) Neurologic signs |  | |  | | | | |  | | | | |  | | | | | |  | | | |  | | | | |  | |
| i) Other |  | |  | | | | |  | | | | |  | | | | | |  | | | |  | | | | |  | |
| j) no disease problem |  | |  | | | | |  | | | | |  | | | | | |  | | | |  | | | | |  | |
| 1. Have any animals been sick in the last 2 weeks? | | | | | | | | | | | | | | 🞏 1 YES  🞏 2 NO | | | | | | | | | | | | | | | |
| 1. If yes, which animal and kind of disease? | | | | | | | | | | | | | | Optional: Use a table of clinical signs by species and key diseases in annex | | | | | | | | | | | | | | | |
| Clinical signs | 1.Cattle | | | 2.Goats | | | | | | 3. Sheep | | | | | | 4. Poultry | | | | | 5. Pigs | | | | 6. Camel | | | | |
| a) Respiratory |  | | |  | | | | | |  | | | | | |  | | | | |  | | | |  | | | | |
| b) Digestive tract/ intestinal |  | | |  | | | | | |  | | | | | |  | | | | |  | | | |  | | | | |
| c) Reproductive |  | | |  | | | | | |  | | | | | |  | | | | |  | | | |  | | | | |
| d) Mastitis |  | | |  | | | | | |  | | | | | |  | | | | |  | | | |  | | | | |
| e) Sudden death |  | | |  | | | | | |  | | | | | |  | | | | |  | | | |  | | | | |
| f) Skin disease |  | | |  | | | | | |  | | | | | |  | | | | |  | | | |  | | | | |
| g) neurologic signs |  | | |  | | | | | |  | | | | | |  | | | | |  | | | |  | | | | |
| h) Other |  | | |  | | | | | |  | | | | | |  | | | | |  | | | |  | | | | |
| 1. Was the disease diagnosed other than by yourself? | | | | | | | | | | | | | | 🞏 1 YES 🞏 2 NO | | | | | | | | | | | | | | | |
| 1. If yes, by whom? | | | | | | | | | | | | | | 🞏 1 Traditional healer  🞏 2 community animal health worker  🞏 3 private veterinarian (Diploma, BVM)  🞏 4 official (governmental) veterinarian  🞏 5 Other (specify)……………………………… | | | | | | | | | | | | | | | |
| 1. What do you do in response to diseases problems? (refer to the recent disease problems mentionned above) | | | | | | | | | | | | | | | | | | | | | | | | | | | | | |
| Activity | | 1.Cattle | | | | | 2.Goats | | | | 3.Sheep | | | | | | 4.Poultry | | | | | 5.Pigs | | | | 6.Camel | | | |
| 1. Use traditional medicine | |  | | | | |  | | | |  | | | | | |  | | | | |  | | | |  | | | |
| 1. Use medicine from the veterinary drug store (self-bought) | |  | | | | |  | | | |  | | | | | |  | | | | |  | | | |  | | | |
| 1. Consult traditional healer | |  | | | | |  | | | |  | | | | | |  | | | | |  | | | |  | | | |
| 1. Consult community animal health worker | |  | | | | |  | | | |  | | | | | |  | | | | |  | | | |  | | | |
| 1. Consult private veterinarian | |  | | | | |  | | | |  | | | | | |  | | | | |  | | | |  | | | |
| 1. Consult Government veterinarian | |  | | | | |  | | | |  | | | | | |  | | | | |  | | | |  | | | |
| 1. Vet applied/left drugs | |  | | | | |  | | | |  | | | | | |  | | | | |  | | | |  | | | |
| 1. Other, please specify | |  | | | | |  | | | |  | | | | | |  | | | | |  | | | |  | | | |
| 1. From the drug categories shown (provide photographs of common veterinary drugs for each of the drug class), how often (how many times) have you used them for the different species in the last 2 months. Alternatively ask to see packaging of commonly used drugs and classify according to list below. | | | | | | | | | | | | | | | | | | | | | | | | | | | | | |
| Drug | | 1 Cattle | | | | 2 Small ruminants | | | | | | 3 Equines | | | | | | 4 Poultry | | | | | 5 Pigs | | | | 6 Camels | | |
| 1 Vaccines | |  | | | |  | | | | | |  | | | | | |  | | | | |  | | | |  | | |
| 2 Antihelmintics (Albendazol, etc.) | |  | | | |  | | | | | |  | | | | | |  | | | | |  | | | |  | | |
| 3 Arachnidicides (ectoparasites) | |  | | | |  | | | | | |  | | | | | |  | | | | |  | | | |  | | |
| 4 Tetracyclines | |  | | | |  | | | | | |  | | | | | |  | | | | |  | | | |  | | |
| 5 Sulphonamides | |  | | | |  | | | | | |  | | | | | |  | | | | |  | | | |  | | |
| 6 Penicillin (and combinations with Penicillin) | |  | | | |  | | | | | |  | | | | | |  | | | | |  | | | |  | | |
| 7 Fluoroquinolones | |  | | | |  | | | | | |  | | | | | |  | | | | |  | | | |  | | |
| 8 Macrolides | |  | | | |  | | | | | |  | | | | | |  | | | | |  | | | |  | | |
| 9 Aminoglycosides | |  | | | |  | | | | | |  | | | | | |  | | | | |  | | | |  | | |
| 10 Other antibiotics (specify) | |  | | | |  | | | | | |  | | | | | |  | | | | |  | | | |  | | |
| 11 Vitamins/Iron supplements | |  | | | |  | | | | | |  | | | | | |  | | | | |  | | | |  | | |
| 12 Other drugs (specify) | |  | | | |  | | | | | |  | | | | | |  | | | | |  | | | |  | | |
| 1. Do you have any particular means to protect animals from disease? | | 🞏 1 YES 🞏 2 NO | | | | | | | | | | | | | | | | | | | | | | | | | | | |
| 1. If yes, how? | | | | | | | | | | | | | | | | | | | | | | | | | | | | | |
| a) Activity | | 1.Cattle | | | 2.Goats | | | | 3.Sheep | | | | | | 3.Poultry | | | | | 4.Pigs | | | | 5.Fish | | | | | 6.Camels |
| b) Fencing | |  | | |  | | | |  | | | | | |  | | | | |  | | | |  | | | | |  |
| c) Not mixing with other herd/flock | |  | | |  | | | |  | | | | | |  | | | | |  | | | |  | | | | |  |
| d) Special feed | |  | | |  | | | |  | | | | | |  | | | | |  | | | |  | | | | |  |
| e) Vet drugs (incl vaccine) | |  | | |  | | | |  | | | | | |  | | | | |  | | | |  | | | | |  |
| 1. Do nothing | |  | | |  | | | |  | | | | | |  | | | | |  | | | |  | | | | |  |
| 1. Other, specify | |  | | |  | | | |  | | | | | |  | | | | |  | | | |  | | | | |  |

| **ANIMAL HEALTH SERVICES** | | | | | | | |
| --- | --- | --- | --- | --- | --- | --- | --- |
| 1. Does the farm have access to professional animal health services? | | | | 🞏 Yes 1  🞏 No 2 | | | |
| 1. If your farm access to animal health services, which ones? | | | | 🞏1 State or government:  🞏a) Fully trained veterinarian (BSc level)  🞏b) Paraveterinarian  🞏c) Other animal health care provider;  🞏d) Don’t know the training or qualification  🞏2 Private full time animal health worker:  🞏a) Fully trained veterinarian (BSc level)  🞏b) Paraveterinarian  🞏c) Other animal health care provider;  🞏d) Don’t know the training or qualification  🞏3 Both state/government and private  🞏a) Fully trained veterinarian (BSc level)  🞏b) Paraveterinarian  🞏c) Other animal health care provider;  🞏d) Don’t know the training or qualification  🞏4 Other (specify)…………………………………………………. | | | |
| 1. If you have access to animal health services, do the animal service include laboratory testing? | | | | 🞏 Yes 1  🞏 No 2 | | | |
| 1. If you have access to laboratory services, do you use it? | | | | 🞏 1 Yes, when needed  🞏 2 Rarely  🞏 3 No | | | |
| 1. If yes, for diagnosis in which species? | | | | | | | |
| 1.Cattle | 2.small ruminants | 3.Equines | 4.Poultry | | 5.Pigs | 6. Camels |  |
| 1. If you don’t use them, why? | | | | 🞏 1 Not available  🞏 2 Not efficient  🞏 3 Too expensive  🞏 4 Would like more  🞏 5 Other (specify)………………………… | | | |
| 1. Is the farm involved in a regular animal health service program, like vaccination campaign etc run by government and/or NGO? | | | | 🞏 1 Yes  🞏 2 No | | | |
| 1. If yes to above, please name | | | | ……………………………… | | | |
| 1. Do you access pharmaceuticals/veterinary drugs? | | | | 🞏 1 Yes  🞏 2 No | | | |
| 1. If yes, which kind of pharmaceuticals/veterinary drugs have you used in the last 4 weeks? (LIST PER SPECIES, PHOTOGRAPH) | | | | List them:……………………………….. | | | |

| **VETERINARY DRUG USE: THE FOLLOWING QUESTIONS FOR EACH SPECIES PRESENT IN THE FARM (ONE DRUG)** | |
| --- | --- |
| 1. Which of the drugs is the most commonly used? (pictures or drug samples) (refer to Q51) | DROP DOWN LIST WITH VET DRUGS |
| 1. Why do you use this drug? | 🞏 1 Disease prevention  🞏 2 Treatment sick animal  🞏 3 Fattening  🞏 4 Other (specify) |
| 1. Via which channel do you access this pharmaceuticals/veterinary drugs | 🞏 1 Private vet  🞏 2 Public/official vet  🞏 3 Animal health worker  🞏4 Veterinary drug store  🞏 5 From human pharmacies  🞏 6 At markets  🞏 7 Feed providers  🞏 8 Other farmers  🞏 9 Via NGOs🞏 10 Other (specify) |
| 1. To which animals do you give the drug? | 🞏 All of the same species  🞏 Sick animals only  🞏 Sick and in contact animals  🞏 Before selling an animal  🞏 Animals newly introduced into herd  🞏 All animals in household |
| 1. How long do you use the drug? | 🞏 As advised  🞏 Until animal(s) cured  🞏 Until package empty  🞏 As long as I can afford  🞏 One time treatment  🞏 Continiously over extended period  Estimated average number days………………. |
| 1. Who adminster the drug? | 🞏 1 Myself  🞏 2 Vet  🞏 Other (specify)……….. |
| 1. How is the drug applied/given? | 🞏 1 Injection  🞏 2 Oral  🞏 3 with feed  🞏 4 with water  🞏 5 on skin  🞏 6 other (specify)……… |

| 1. Do you get advice how to use the vet drugs? | 🞏 Yes 1  🞏 No 2 |
| --- | --- |
| 1. If yes to above, via which channel? | 🞏 1 from the veterinarians  🞏 2 from the animal health worker  🞏 3 from pharmacies or markets  🞏 4 from other farmers  🞏 5 via the feed provider  🞏 6 from the package/label of he pharmaceutical  🞏 7 Other, state who……….  🞏 8 No, own judgment |
| 1. When using veterinary drugs, whose instructions (kind, dose, length of treatment) do you follow: | 🞏1 The Veterinarian’s  🞏2 The animal health worker’s  🞏3 The pharmacy’s  🞏4 The feed company’s  🞏5 Other farmer’s  🞏6 My own judgement  🞏7 Other`s(specify)………………………………. |

| **USE OF ANTIBIOTICS** | |
| --- | --- |
| 1. What does vaccination do?   (multiple answers possible) | 🞏1 Cure sick animals  🞏2 Prevent animals from becoming sick  🞏3 Cure sick animals and prevent animals from becoming sick  🞏4 Fattening |
| 1. What do antibioticls do? (multiple answers possible) | 🞏1 Cure sick animals  🞏2 Prevent animals from becoming sick  🞏3 Cure sick animals and prevent animals from becoming sick  🞏4 Fattening |
| 1. If the subject understands what antibiotics (option 1 and 3) are based on the two questions above continue as follows: | |
| 1. Do you consume milk, from animals who were just treated with antimicrobials. | 🞏 1 Yes  🞏 2 No |
| 1. If No to the above, for how long time should those products be avoided (open) (in days) | ……………………………….. |
| 1. Do you consume eggs from animals who were just treated with antimicrobials. | 🞏 1 Yes  🞏 2 No |
| 1. If No to the above, for how long time should those products be avoided (open) (in days) | ……………………………….. |
| 1. Do you consume meat from animals who were just treated with antimicrobials. | 🞏 1 Yes  🞏 2 No |
| 1. If No to the above, for how long time should those products be avoided (open) (in days) | ……………………………….. |

| 1. Have you experienced situations where drugs did not work | | | Yes, frequently  Yes, sometimes  No, never | | | | | |
| --- | --- | --- | --- | --- | --- | --- | --- | --- |
| 1. If you have experience with drug failure (YES to Q71), which drugs did not work? | | | | | | | | |
| Drug on pictures shown | 1.Cattle | 2.Poultry | | 3.Pigs | 4.Small ruminants | 5.Equine | 6.Camels |  |
| Drug list |  |  | |  |  |  |  |  |
| Drug list |  |  | |  |  |  |  |  |
| Drug list |  |  | |  |  |  |  |  |
| 1. If the veterinary drug do not work, do you know why? | | | ………………………………………………………….. | | | | | |
| 1. What do you do with expired veterinary drugs? | | | 🞏1 Dispose off  🞏2 Return to pharmacy  🞏3 Give to other farmer  🞏4 Use for intended treatment  🞏5 Nothing  🞏6 Other (specify) | | | | | |
| 1. **FOR EACH SPECIES:** What was your total expenditure in drugs during the last year in local currency (put 0 if none) | | | 🞏1 Dewormer […………………]  🞏2 Vaccination […………………]  🞏3 Antibiotics […………………]  🞏4 Acaracides […………………]  🞏5 Vitamins | | | | | |

**NB: Note that the GIS may take some time to load approximately 5 minutes however they work**

**List of annexes**

1. List of pictures of drugs, organised into drug classes as specified in question 40.
2. List of clinical signs by syndrome
3. Groups drug used by de-wormers, vaccines, AB, vitamins
4. List of social events and festive seasons with the months they happen
